# Physics-informed neural network for predicting *in vacuo* vocal fold eigenmodes: A proof of concept study

**DOI:** 10.1121/10.0043248

**Published:** 2026-04-06

**Authors:** Mohd Ethar M Al Khasawneh, Michael Döllinger, Zhaoyan Zhang

**Affiliations:** 1 Division of Phoniatrics and Pediatric Audiology, Department of Otorhinolaryngology Head & Neck Surgery, University Hospital Erlangen, Friedrich-Alexander-University Erlangen-Nürnberg, Waldstrasse 1, 91054 Erlangen, Germany; 2 Department of Head and Neck Surgery, University of California, Los Angeles, 31-24 Rehabilitation Center, 1000 Veteran Avenue, Los Angeles, California 90095-1794, USA

## Abstract

This study investigates a machine-learning approach for real-time computation of *in vacuo* vocal fold eigenmodes. A physics-informed neural network is trained to predict eigenmodes and eigenfrequencies by integrating the governing equations of vocal fold dynamics. The proposed architecture predicts physically consistent modal shapes and frequency estimates, showing strong agreement with finite-element method results for lower-order modes, achieving mean relative errors below 6% in eigenfrequency prediction and cosine correlation values approaching 1 in eigenmode prediction, while prediction accuracy declines for higher-order modes. These findings demonstrate that the physics-informed neural network provides an accurate and efficient real-time computation of *in vacuo* vocal fold eigenmodes.

## 1. Introduction

Human voice production arises from interactions between airflow and vocal fold vibrations (Zhang, 2016). Accurate modeling of vocal fold vibration is crucial for clinical assessment and diagnosis of voice disorders. Computational tools that reliably characterize the effects of physiological changes on vocal fold vibrational behavior are essential for guiding treatment planning, thereby supporting restoration of the mechanical conditions required for normal phonation (Zhang, 2016). Due to the high computational costs in resolving the complex fluid-structure-acoustic interaction during voice production, reduced-order models based on eigenmode superposition have been developed to reduce the computational costs (Zhang, 2015, 2017b,a). In this eigenmode-based approach, the natural eigenmodes of the vocal folds are first calculated, often using the finite-element method (FEM), and subsequently used as a basis function to approximate vocal fold vibration in the interaction with airflow. The natural frequencies and eigenmodes of the vocal folds characterize their vibration behavior and determine how vocal fold tissue motion interacts with aerodynamic forces during phonation (Zhang, 2016). Experimental studies have demonstrated strong correspondence between observed vocal fold vibration patterns and structural eigenmodes (Berry and Titze, 1996), indicating that vocal fold motion can be accurately described by the superposition of the eigenmodes (Titze, 1976; Zhang, 2015; Titze and Strong, 1975; Tokuda *et al.*, 2007; Svec *et al.*, 2000).

Although the FEM method provides high accuracy in computing vocal fold eigenmodes, it is computationally expensive and does not allow real-time analysis (Zhang, 2015, 2017a,b). In this study, we aim to reduce the computational cost associated with conventional numerical approaches in solving the eigenvalue problem and achieve a real-time inference by using a physics-informed neural network (PINN). This approach is employed because it directly integrates the governing equations of vocal fold vibration together with the associated boundary conditions within the training process, ensuring physically consistent predictions (Raissi *et al.*, 2019). In addition, selected data obtained from FEM simulations are used to guide the network training and improve prediction accuracy. In this proof of concept study, we will show that this approach, when trained using sufficient data, has the potential for real-time prediction of the *in vacuo* vocal fold eigenmodes, thus significantly reducing the time for computational voice production simulation.

## 2. Method

### 2.1 Governing equation

The vocal fold eigenvalue problem is formulated using the body–cover model (Zhang, 2017a,b). Although vocal fold tissue exhibits nonlinear and anisotropic behavior (Zhang, 2016, 2017a), each layer is approximated as a nearly incompressible, transversely isotropic, linear elastic material with the plane of isotropy perpendicular to the anterior–posterior direction. Under the assumption of linear elasticity (Titze, 1976; Titze and Talkin, 1979; Zhang, 2015), the stress–strain relation follows Hooke's law (Lautrup, 2011, Chap. 11),

$$\sigma_{ij}=C_{\text{ijk}\ell }\;\varepsilon_{k\ell },$$ (1)

where $C_{\text{ijk}\ell }$ is the fourth-order elasticity tensor. The Cauchy strain tensor is defined as

$$\varepsilon_{ij}=\frac{1}{2}\left(\nabla_{i}u_{j}+\nabla_{j}u_{i}\right).$$ (2)

Mechanical equilibrium is given by Lautrup (2011, Chap. 12),

$$f_{i}^{*}=f_{i}+\sum_{j}\nabla_{j}\sigma_{ij}=0,$$ (3)

where $f_{i}^{*}$ denotes the effective force density and $f_{i}$ the external body force. Substituting Eqs. (1) and (2) into Eq. (3), neglecting external body forces under the *in vacuo* assumption (Berry and Titze, 1996; Zhang, 2015, 2017b), and subsequently applying Newton's second law to an infinitesimal material element expressed in terms of force density yields Navier's equation of motion (Lautrup, 2011, Chap. 14):

$$\rho \;\frac{\partial^{2}u_{i}}{\partial t^{2}}=\nabla_{j}(C_{\text{ijk}\ell }\;\frac{1}{2}\left(\nabla_{k}u_{\ell }+\nabla_{\ell }u_{k}\right)).$$ (4)

Expressing the displacement field in harmonic form transforms the time-dependent problem into a time-independent formulation (Lautrup, 2011, Chap. 14):

$$u(x,t)=u(x)e^{-i\omega t},\;\frac{\partial^{2}u_{i}}{\partial t^{2}}=-\omega^{2}u_{i}(x)e^{-i\omega t}.$$ (5)

Substituting the harmonic displacement representation and its second derivative given in Eq. (5) into the time-domain Navier–Cauchy equation results in the frequency-domain governing equation

$$-\rho \;\omega^{2}u_{i}=\nabla_{j}\left[C_{\text{ijk}\ell }\;\frac{1}{2}\left(\nabla_{k}u_{\ell }+\nabla_{\ell }u_{k}\right)\right].$$ (6)

Equation (6) serves as the governing equation for the PINN. The corresponding residual is defined as

$$R_{i}(x;\theta )=\nabla_{j}[C_{\text{ijk}\ell }\;\frac{1}{2}\left(\nabla_{k}u_{\ell }(x;\theta )+\nabla_{\ell }u_{k}(x;\theta )\right)]+\rho \;\omega^{2}u_{i}(x;\theta ),$$ (7)

which is enforced to vanish at the collocation points within the computational domain. Here, u(x;θ) denotes the displacement field predicted by the neural network, *x* represents the spatial collocation points, and θ collects the trainable network parameters.

### 2.2 Boundary conditions

The vocal fold model is subject to Dirichlet, Neumann, and interface boundary conditions to suppress rigid-body motion, enforce traction-free surfaces, and ensure mechanical continuity between the body and cover layers. Dirichlet conditions prescribe zero displacement on mechanically fixed surfaces (lateral, anterior, and posterior) (Faust *et al.*, 2024), while Neumann conditions impose zero traction on the free surfaces (medial, superior, and inferior) (Faust *et al.*, 2024). The two-layer structure is assumed to be perfectly bonded at the internal interface. Accordingly, displacement compatibility and traction equilibrium are enforced, consistent with standard elasticity interface conditions for weak discontinuities (van den Boom *et al.*, 2019). These conditions ensure continuous stress transmission and mechanical equilibrium across the body–cover interface.

### 2.3 Rayleigh quotient

The Rayleigh quotient has previously been incorporated into PINNs for eigenvalue problems (Kovacs *et al.*, 2022). Building on this approach, we adopt a Rayleigh quotient formulation tailored to the linear elasticity model of the vocal folds, where the squared angular frequency $\omega^{2}$ is expressed as the ratio of elastic strain energy *V*[*u*] to kinetic energy *T*[*u*] evaluated over the domain (Chan *et al.*, 2011),

$$\lambda_{\text{RQ}}=\omega^{2}=\frac{V[u]}{T[u]}=\frac{\int_{V}C_{\text{ijkl}}\;\partial_{j}u_{i}\;\partial_{\ell }u_{k}\;dV,}{\int_{V}\rho \;u_{i}u_{i}\;dV,},$$ (8)

where *u* denotes the displacement field, $C_{\text{ijkl}}$ is the elasticity tensor, and ρ is the mass density. In the current study, the vocal fold consists of body and cover layers with distinct material properties. The Rayleigh quotient is therefore evaluated over both regions using collocation points corresponding to each layer. The displacement field predicted by the neural network is substituted directly into this formulation to obtain the eigenfrequency associated with each mode.

### 2.4 Loss function

In PINNs, the loss function combines data-driven terms with physics-based constraints derived from governing equations and boundary conditions (Raissi *et al.*, 2019). The network is trained by minimizing a composite loss defined as

$$L_{\text{Total}}=L_{\text{Physical}}+L_{\text{Data}-\text{driven}}.$$ (9)

The physical component enforces the governing equations and boundary conditions, ensuring physically consistent network predictions. The term $L_{\text{governing}-\text{equation}}$ penalizes the residual of the Navier–Cauchy Eq. (7). All physical terms are formulated using mean squared error, and the physical loss is given by

$$L_{\text{physical}}=L_{\text{governing}-\text{equation}}+L_{\text{Dirichlet}}+L_{\text{Neumann}}+L_{\text{interface}}.$$ (10)

The data-driven loss contains eigenvalue and mode shape consistency terms:

$$L_{\text{Data}-\text{driven}}=L_{\text{Eigenvalue}}+L_{\text{ cos}-\text{correlation}}.$$ (11)

The eigenvalue loss penalizes discrepancies between the Rayleigh quotient eigenvalue $\lambda_{\text{RQ}}$ and the reference eigenvalue $\lambda_{\text{True}}$. Although the network does not directly predict eigenvalues, this term enforces consistency between the eigenvalues derived from the predicted displacement field and the reference solution and is formulated as

$$L_{\text{Eigenvalue}}=\frac{{\left(\lambda_{\text{RQ}}-\lambda_{\text{True}}\right)}^{2}}{{\left(\lambda_{\text{True}}\right)}^{2}}.$$ (12)

Direct pointwise metrics such as mean squared error are unsuitable for comparing eigenmodes because modal amplitudes are not uniquely defined. Instead, cosine correlation is employed, which measures similarity based on the angle between two vectors and is invariant to vector magnitude (Xia *et al.*, 2015). Values approaching 1 indicate strong agreement. In the current work, the mode shape corresponds to the displacement field predicted by the neural network. The cosine correlation loss is defined as

$$L_{\text{ cos}-\text{correlation}}=1-\underset{\text{ cos}(\theta )\text{ cosine correlation}}{\underset{︸}{\frac{u_{\text{Pred}}\cdot u_{\text{True}}}{\left||u_{\text{Pred}}||\;||u_{\text{True}}|\right|}}},$$ (13)

where $u_{\text{Pred}}$ and $u_{\text{True}}$ denote the predicted and reference displacement vectors, respectively.

### 2.5 Dataset and preprocessing

The body–cover vocal fold biomechanical model used to train the PINN follows the formulation in Zhang (2017a,b). The geometric domain is fixed, with medial surface thickness T=4.5 mm, body depth $D_{b}=60\text{ mm}$, cover depth $D_{c}=15\text{ mm}$, and anterior–posterior (AP) length L=17 mm. A uniform density of $\rho =1030\;{\text{kg}/m}^{3}$ is used throughout the domain. Material variation is introduced through the transverse Young's modulus $E_{t}\in \{1,2,4\}\text{ kPa}$ and the AP shear moduli of the body and cover layers, $G_{\text{AP}}\in \{1,10,20,30,40\}\text{ kPa}$, with $E_{\text{AP}}=4G_{\text{AP}}$. All parameter combinations yield 75 material configurations. For each configuration, eigenfrequencies and eigenmodes are computed using finite-element simulations. The dataset is split into 60 (80%) for training and 15 (20%) for validation. Prior to training, the elasticity matrix C is computed for each material configuration defined by $E_{t}$, $G_{\text{APcover}}$, and $G_{\text{APbody}}$, following the isotropically compressible transversely isotropic formulation in Itskov and Aksel (2002). The precomputed elasticity matrix C is incorporated into the governing equations, Rayleigh quotient, and boundary and interface conditions during training. Collocation points are sampled to enforce the physical constraints, and the body-to-cover volume ratio is used to ensure consistent sampling and accurate integration of strain and kinetic energy contributions in the Rayleigh quotient.

### 2.6 Network architecture and training strategy

The proposed model combines a PINN with a sinusoidal representation network (SIREN) backbone. SIREN is selected for its ability to represent high-frequency spatial features and compute smooth higher-order derivatives via automatic differentiation, which is essential for enforcing governing-equation residuals and boundary constraints (Sitzmann *et al.*, 2020). The network inputs consist of spatial coordinates $x={\left(x,y,z\right)}^{⊤}$ and material parameters $\theta ={\left(E_{t},G_{\text{APbody}},G_{\text{APcover}}\right)}^{⊤}$. The material parameters are encoded through two fully connected layers with sigmoid linear unit (SiLU) activations to capture nonlinear relationships between material properties and modal behavior. The encoded parameters are then concatenated with the spatial coordinates before being passed to the SIREN backbone. The SIREN consists of four hidden layers with 128 neurons per layer and sinusoidal activations with frequency scaling ω=5, enabling accurate representation of fine spatial features and reliable computation of the derivatives required by the physical loss. Training is performed independently for each vibration mode to avoid mode mixing. For each mode *k*, the network predicts the displacement field ${\hat{u}}_{k}$, from which the eigenvalue $\lambda_{\text{RQ}}$ is computed via the Rayleigh quotient Eq. (8), ensuring physically consistent coupling between displacement and frequency. The physics-informed loss Eq. (10) enforces the Navier–Cauchy equation Eq. (7) boundary and interface conditions. Additional supervision is provided through penalty terms on eigenvalue discrepancy Eq. (12) and mode-shape alignment via cosine correlation Eq. (13).

## 3. Results

**Fig. 1.**
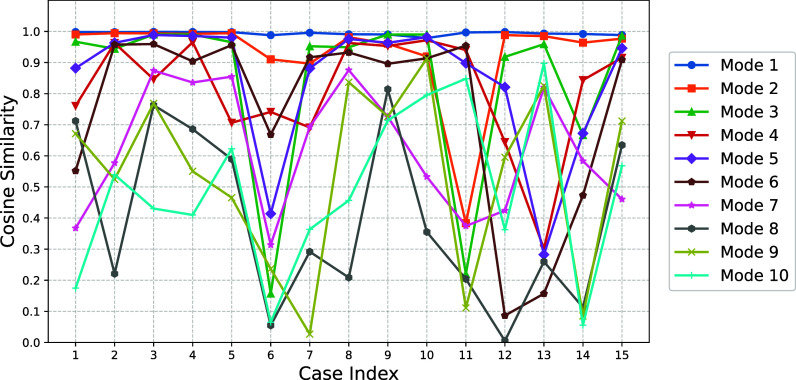
Comparison between predicted and true eigenmodes for modes 1–10 across 15 validation cases, quantified using cosine similarity metrics.

**Fig. 2.**
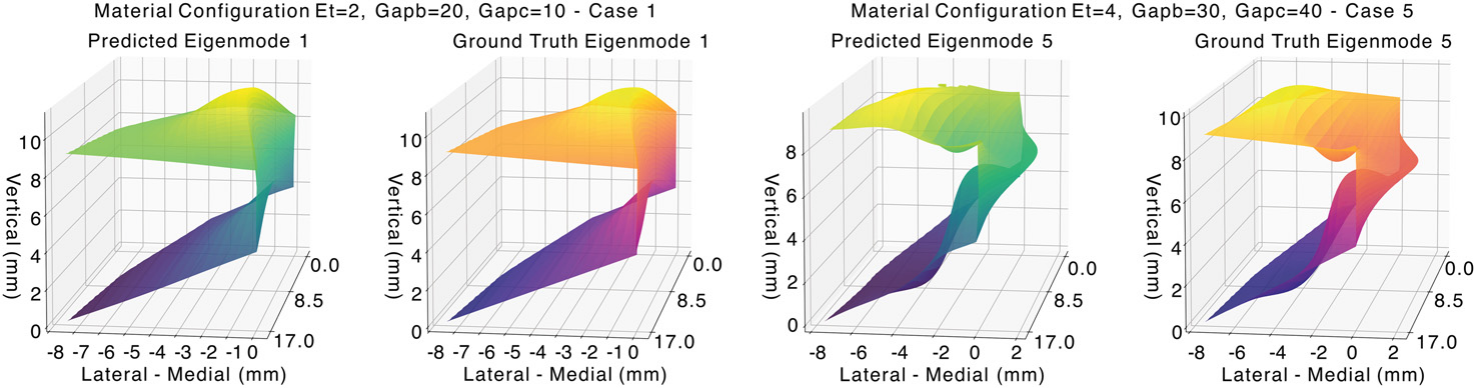
Three-dimensional visualization of predicted and reference (ground truth) vocal fold eigenmodes for selected validation cases. Predicted and ground-truth mode shapes are shown for eigenmodes 1 and 5 under different material configurations.

**Fig. 3.**
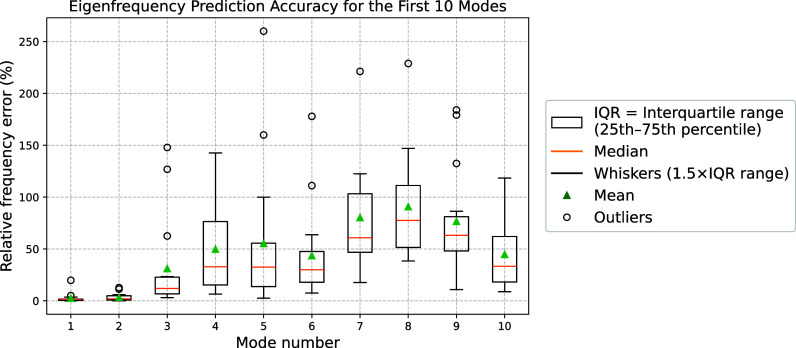
Statistical distribution of relative eigenfrequency prediction errors across 15 validation cases for the first ten vibration modes.

Table 1 summarizes the validation performance of the proposed PINN for the first ten vocal fold modes. The results demonstrate accurate prediction of eigenmodes and eigenfrequencies for lower-order modes, while accuracy decreases with increasing mode order. Higher cosine correlation is generally associated with lower eigenfrequency error when evaluated through the Rayleigh quotient. Figure 1 further illustrates strong agreement between predicted and reference mode shapes for lower-order modes, while higher-order modes exhibit reduced alignment. Figure 2 presents three-dimensional visualizations of predicted and reference eigenmodes for two representative material configurations from the validation set. The left pair corresponds to eigenmode 1 under material configuration case 1, while the right pair shows eigenmode 5 under material configuration case 5. The results demonstrate qualitative agreement between predicted and reference mode shapes, consistent with the cosine similarity reported in Fig. 1. Figure 3 shows the distribution of eigenfrequency predictions across validation cases, with lower-order modes displaying narrower spreads and higher-order modes showing greater variability.

**Table 1.** PINN performance for the first ten vocal fold modes. Eigenfrequencies are shown for validation case 1 with material configuration ( $E_{t}=2\text{ kPa}$, $G_{\text{AP},\text{body}}=20\text{ kPa}$, $G_{\text{AP},\text{cover}}=10\text{ kPa}$). Mean predicted (Pred.) eigenfrequency and eigenmode metrics are computed across the validation dataset after 3000 training epochs. Rel., relative; corr., correlation.

Mode	True ω (Hz)^a^	Pred. ω (Hz)^a^	Rel. error (%)^a^	Cosine corr.^a^	Mean Rel. error (%)^b^	Mean cosine corr.^b^
1	90.60	90.79	0.21	0.99	4.78	0.99
2	96.17	97.27	1.14	0.98	5.78	0.92
3	110.23	125.68	14.01	0.94	22.19	0.84
4	116.31	123.86	6.55	0.96	44.60	0.81
5	122.37	140.38	14.71	0.97	48.61	0.84
6	136.26	177.11	29.98	0.93	44.41	0.74
7	139.93	212.45	51.82	0.87	79.34	0.62
8	145.86	201.84	38.37	0.20	89.07	0.39
9	148.36	217.49	46.59	0.83	76.47	0.53
10	157.25	174.40	10.90	0.45	45.11	0.48

^a^ Metrics computed for validation case 1.

^b^ Mean values computed across the validation dataset.

Table 2 highlights differences in modal behavior across material configurations through cosine correlation analysis of reference eigenmodes. The first and second modes exhibit the highest correlation values (mean of approximately 0.66–0.67, with higher medians), indicating that their spatial deformation patterns remain highly consistent despite variations in material properties. This structural stability reduces learning complexity and contributes to the strong eigenfrequency and mode-shape predictions observed for lower-order modes. In contrast, higher-order modes exhibit significantly lower cosine correlation values, reflecting greater sensitivity of their spatial patterns to material variation. As a result, different material configurations may produce substantially distinct higher-order mode shapes, increasing the complexity of the underlying modal space. The current training dataset therefore provides limited coverage of these diverse patterns, making generalization more challenging for the PINN. The reduced accuracy in eigenfrequency and mode-shape predictions for higher-order modes consequently suggests that additional training data particularly targeting higher-order modal behavior would be required to enhance the learning capacity and predictive robustness of the PINN.

**Table 2.** Summary statistics of cosine correlation between the reference (ground truth) eigenmode displacement fields across the training data set material configurations.

	Cosine correlation	
Mode	Median	Mean
1	0.6907	0.6624
2	0.7825	0.6717
3	0.2592	0.4064
4	0.4482	0.4556
5	0.2169	0.3080
6	0.1810	0.3291
7	0.1757	0.2945
8	0.1545	0.2300
9	0.1219	0.2115
10	0.2039	0.2767

## 4. Discussion and conclusions

The proposed PINN architecture provides real-time computation of vocal fold eigenmodes and associated eigenfrequencies, with particularly strong predictive performance for lower-order modes. The results show that the first two modes are predicted with high accuracy in both eigenfrequency and mode-shape alignment, reflecting the relative stability of their spatial deformation patterns across material configurations. In contrast, predictive performance decreases for higher-order modes, where increased spatial complexity and greater sensitivity to material variation introduce additional challenges. This behavior is consistent with the statistics reported in Table 2. A primary limitation arises from the limited training data available for higher-order modes. The current dataset may be insufficient to capture the increased spatial complexity of these modes across diverse material configurations. Expanding the training dataset to include additional material combinations would improve the network's ability to learn the complex spatial patterns associated with higher-order modes and, consequently, enhance predictive accuracy. A secondary limitation concerns the formulation of the cosine similarity loss. Although the governing equations, boundary conditions, and Rayleigh quotient are enforced throughout the volumetric domain, cosine similarity is evaluated only on surface points. While this reduces computational cost during training, it may limit the network's ability to capture internal deformation structures that become more pronounced in higher-order modes. Extending the similarity constraint to volumetric displacement fields may further improve representation of complex modal patterns. Another limitation is the independent training of each eigenmode using separate PINN models. This strategy avoids mode mixing but differs from classical modal analysis, where orthogonality of mass and stiffness matrices enforces modal separation across the entire basis (Meirovitch, 2001, Chap. 7.7). The absence of explicit orthogonality constraints may contribute to reduced modal separation and eigenfrequency inaccuracies observed in intermediate and higher-order modes. Incorporating orthogonality loss terms could improve modal ordering and overall robustness.

The current architecture is restricted to a single vocal fold geometry, limiting generalization across anatomical variability. Extending the framework to accommodate geometric variations would enhance its applicability to patient-specific modeling and broader clinical scenarios. Future work will focus on scalable architectures capable of predicting multiple eigenmodes within a unified model while incorporating geometric variability. A major advantage of the proposed architecture lies in its computational efficiency at inference. For a single material configuration, eigenmode and eigenfrequency computation using the commercial FEM software (comsol) required approximately 9 min on a system equipped with an Apple M1 Pro processor (Apple, Cupertino, CA) and 16 GB of memory. In contrast, evaluation using the trained PINN on the same system required approximately 1.6 s, representing a substantial reduction in computational cost and enabling real-time modal analysis. Although network training required approximately 30 h on a single Nvidia V100 graphics processing unit (GPU) (Nvidia, Santa Clara, CA) to learn three eigenmodes, this cost is incurred only once during offline training. Once trained, the model provides rapid predictions, making it well suited for clinical applications that require repeated or interactive assessments. Overall, the results demonstrate that PINNs provide a computationally efficient alternative to conventional finite-element eigenanalysis for vocal fold modal characterization. While predictive accuracy for higher-order modes can be further improved through increased data diversity and enhanced constraint formulations, the present framework establishes a foundation for real-time, physics-consistent modal analysis of vocal fold dynamics.

## Data Availability

The data that support the findings of this study are available from the corresponding author upon reasonable request.
